# Supplementary material for: APP Knock-In Mice Produce E22P-Aβ Exhibiting an Alzheimer’s Disease-like Phenotype with Dysregulation of Hypoxia-Inducible Factor Expression
Source: Int J Mol Sci. 2022 Oct 31;23(21):13259. doi: 10.3390/ijms232113259 (PMC9654501; doi:10.3390/ijms232113259)
Supplement: Supplementary file 1 [file ijms-23-13259-s001.zip › ijms-1968200-supplementary.pdf]

International Journal of *Molecular Sciences*

MDPI

Supplementary Material

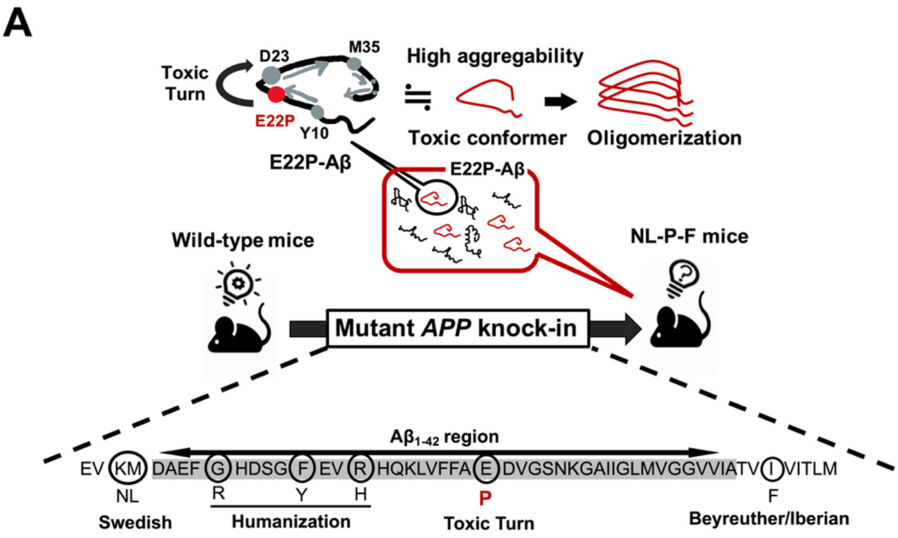

Figure S1. To construct the APP knock-in mice, the sequence for Aβ and the surrounding mutation sites related to gene mutation of familial AD were substituted. Swedish double mutations (K670N substitution and M671L substitution), Beyreuther/Iberian (I716F substitution) and Toxic Turn (E693P substitution) mutations were introduced. Furthermore, the Aβ sequence was humanized by introducing mutations leading to the substitutions G676R, F681Y, and R684H. E22P-Aβ has an intramolecular turn at positions 22–23, forming a toxic conformer. Toxic conformers are highly aggregative and promote Aβ oligomerization. Under physiological conditions, *APP*$^{NL\text{-}P\text{-}F/NL\text{-}P\text{-}F}$ (NL-P-F) mice exhibit phenotypes characterized by toxic conformers with production of E22P-Aβ. (A) NL-P-F mice are generated by knock-in technique of the mutant *APP* gene and produce Aβ oligomers by toxic conformers.

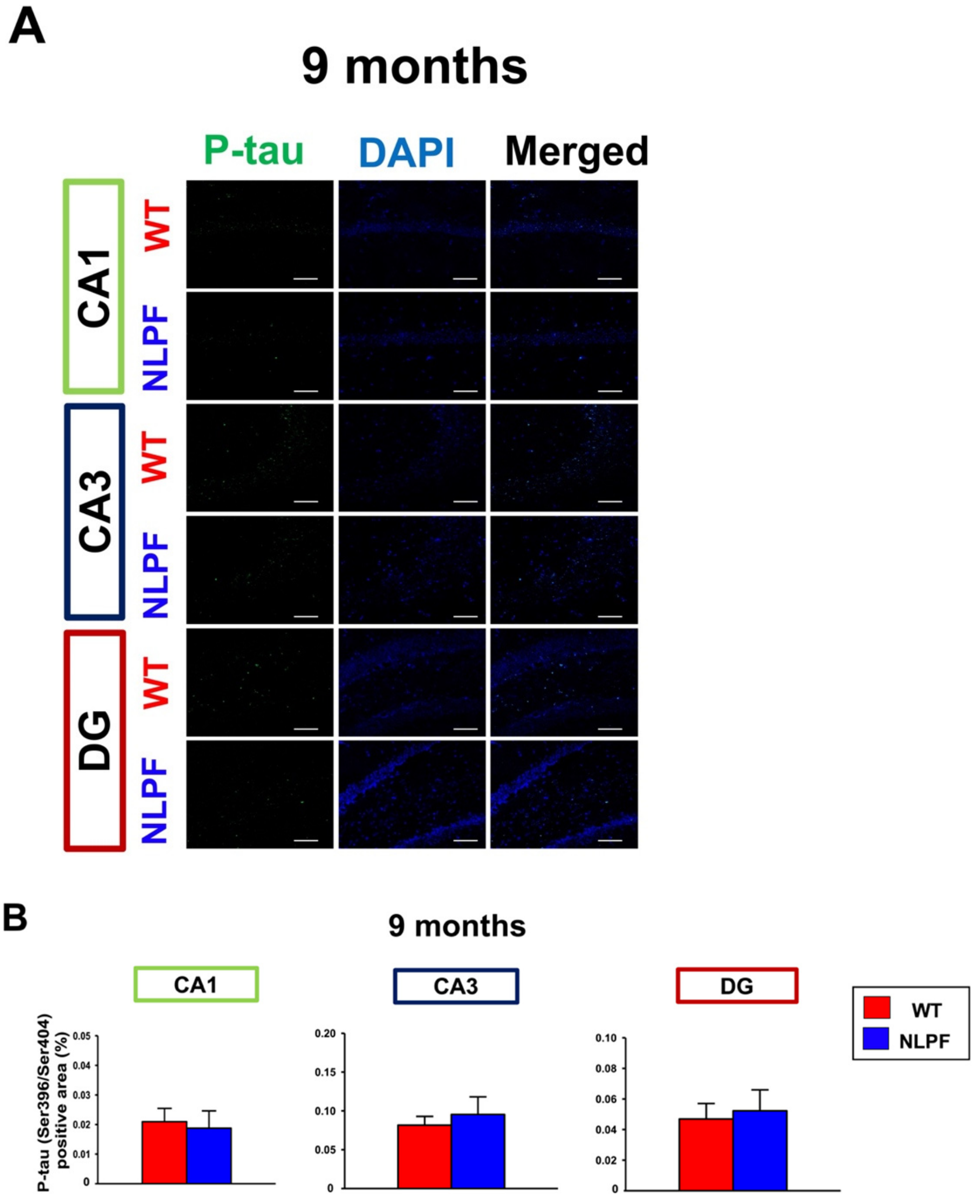
A
9 months
P-tau
DAPI
Merged
CA1
CA3
DG
WT
NLPF
B
9 months
CA1
CA3
DG
WT
NLPF
P-tau (Ser396/Ser404) positive area (%)
0.05
0.04
0.03
0.02
0.01
0
0.20
0.15
0.10
0.05
0
0.10
0.08
0.06
0.04
0.02
0

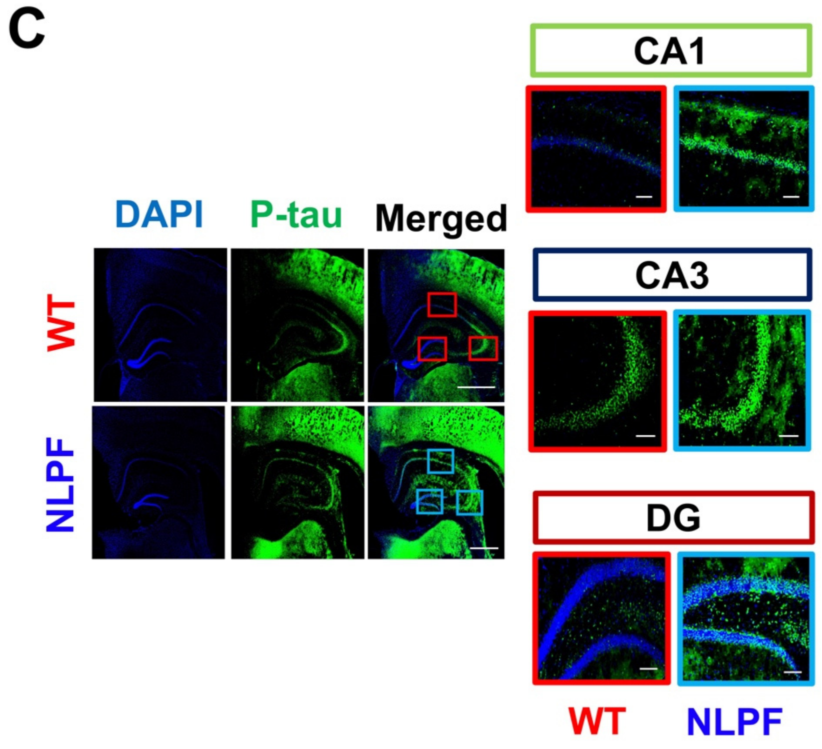

Figure S2. The phosphorylated tau-positive area in the hippocampal CA1, CA3, and DG (A–B) at nine months and (C) twelve months in wild-type (WT) and NL-P-F (NLPF) mice. (A) Representative confocal images. Scale bar indicates 50 μm. (B) The phosphorylated tau-positive area (% of total area). (C) Representative fluorescent images. The scale bar indicates 1 mm (50 μm in enlarged images) WT and NLPF (*n* = 6). Values indicate the mean ± SEM.

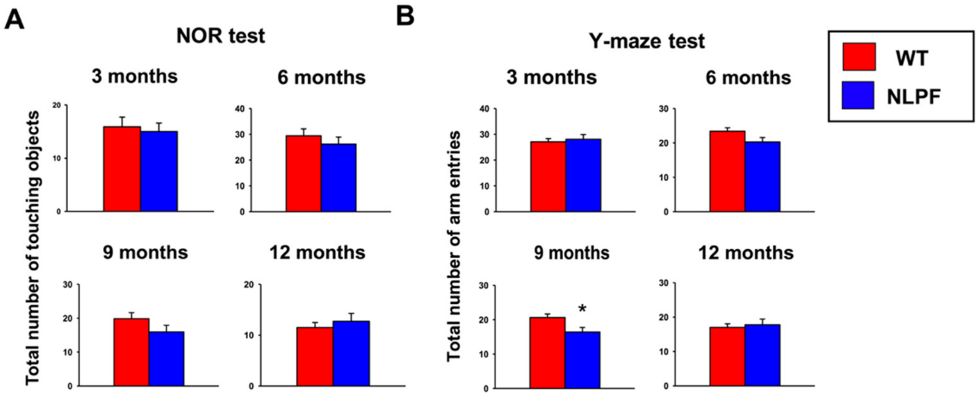

Figure S3. From three to twelve months, the total number of touches in the NOR test and entries in the Y-maze test were almost the same. (A) Total number of times mice touched familiar and novel objects in the NOR test. (B) Total number of times mice entered each arm in the Y-maze test. Values indicate the mean ± SEM. *$P < 0.05$, compared with age-matched WT, two-way ANOVA post hoc Holm-sidak Tukey's test.

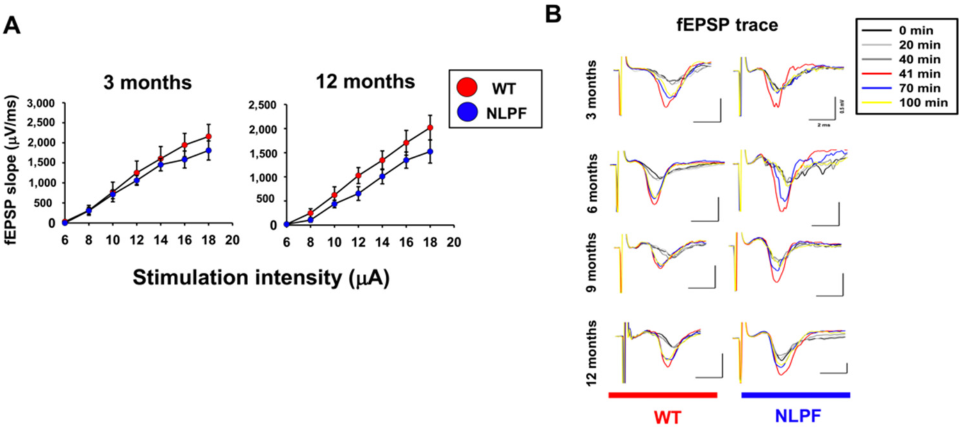

Figure S4. From three to twelve months, Input-output curves were measured at hippocampal CA1 synapses and representative fEPSP trace of LTP measurement are shown. (A) Input-Output curve. WT and NLPF (*n* = 9) at three months, WT (*n* = 11) and NLPF (*n* = 10) at twelve months. (B) Representative fEPSP traces of LTP measurement (black: 0 min, gray: 20 min, dark gray: 40 min, red: 41 min, blue: 70 min, and yellow: 100 min). Values indicate the mean ± SEM.

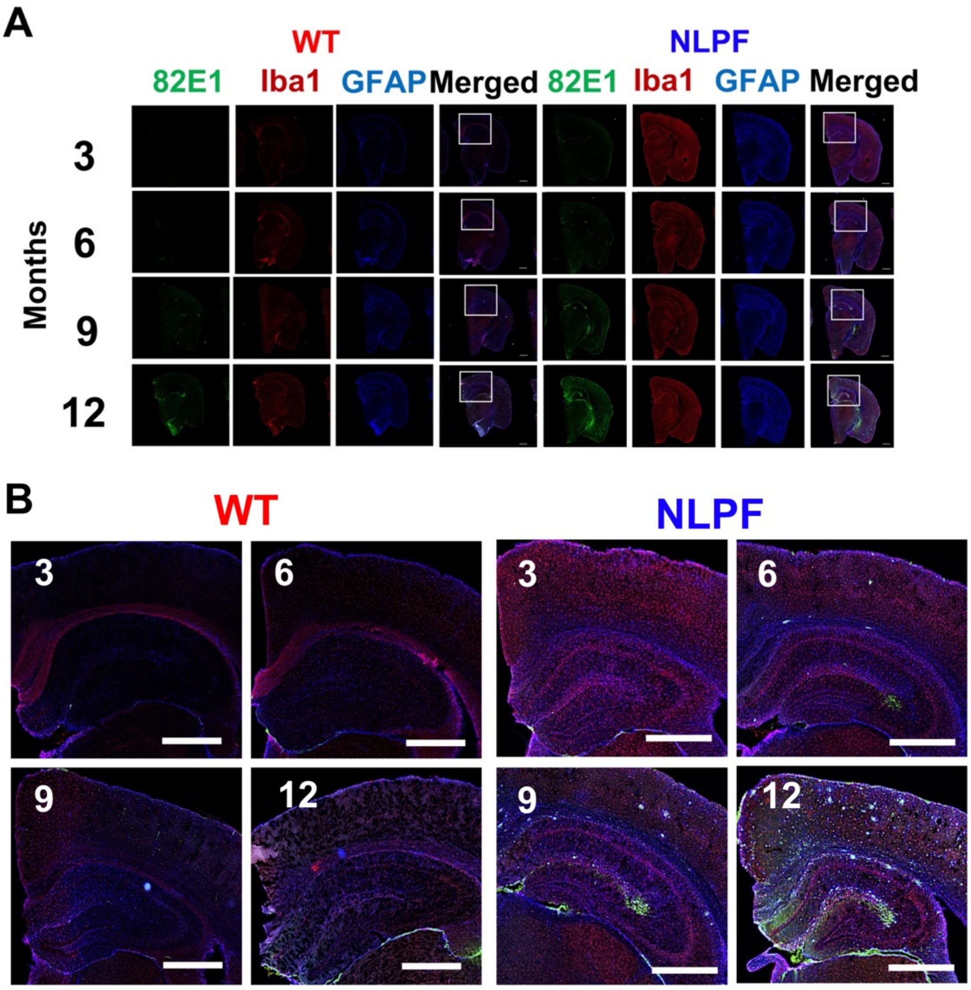

Figure S5. Triple stained images of coronal brain sections including the hippocampal region. Increased Iba1 and GFAP fluorescence was detected in NL-P-F mice from three to twelve months. (A) Aβ deposition and glial cell activation in NL-P-F mice. Microglia and astrocytes were immunostained with antibodies for Iba1 and GFAP, respectively. Aβ deposition was detected by

82E1. (B) Enlarged images of the square surrounded by the white line in (A). Scale bar indicates 1 mm.

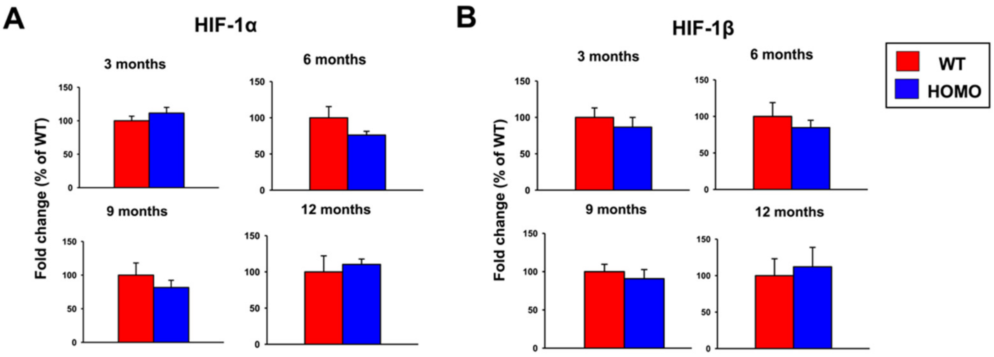

Figure S6. Relative gene expression levels of (A) HIF-1α and (B) HIF-1β of hippocampal tissue of NL-P-F mice from three to twelve months. Gene expression levels were not significantly different between wild-type and NL-P-F mice. (A) WT (n = 5–6), NLPF (n = 6). (B) WT (n = 5–6), NLPF (n = 6). Values indicate the mean ± SEM.

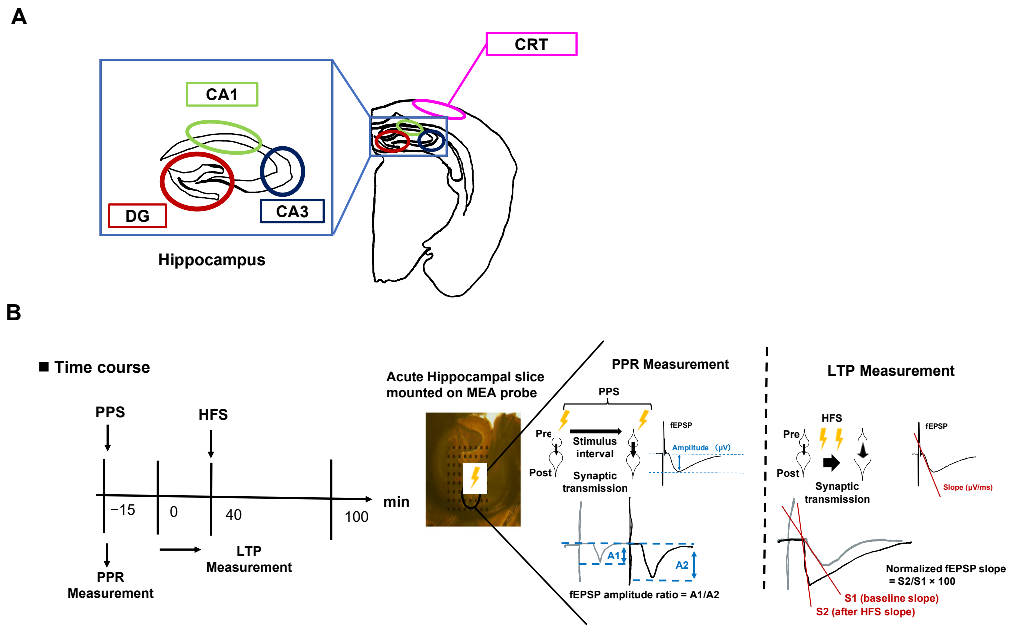

Figure S7. (A) Staining area of coronal sections of brain hemisphere were shown. Hippocampal CA1, CA3, DG, and cortex (CRT) were analyzed by Immunohistochemistry. (B) Time course and method to calculate PPR and LTP of the electrophysiological recording.

Table S1. Detailed information on statistical analysis.

Fig.	F value	P-value	Statistical method
1A	Genotype: (1, 29) = 216.755<br>Month: (3, 29) = 30.330<br>Genotype x Month: (3, 29) = 30.469	Genotype: < 0.001<br>Month: < 0.001<br>Genotype x Month: < 0.001<br>NLPF vs. WT<br>3 months: 0.004<br>6 months: 0.002<br>9 months: < 0.001<br>12 moths: < 0.001	2 way ANOVA post hoc holm-sidak Tukey's test
1B	Genotype: (1, 28) = 38.430	Genotype: < 0.001	2 way ANOVA post hoc holm-sidak Tukey's test

	Month: (3, 28) = 13.397<br>Genotype x Month: (3, 28) = 13.395	Month: < 0.001<br>Genotype x Month: < 0.001<br>3 months: 0.932<br>6 months: 0.661<br>9 months: < 0.001<br>12 moths: < 0.001	
2B-CA1	Genotype: (1, 48) = 37.473<br>Month: (3, 48) = 18.341<br>Genotype x Month: (1, 48) = 18.378	Genotype: < 0.001<br>Month: < 0.001<br>Genotype x Month: < 0.001<br>NLPF vs. WT<br>3 months: 0.982<br>6 months: 0.647<br>9 months: 0.014<br>12 moths: < 0.001	2 way ANOVA post hoc holm-sidak Tukey's test
2B-DG	Genotype: (1, 48) = 46.633<br>Month: (3, 48) = 22.669<br>Genotype x Month: (3, 48) = 22.843	Genotype: < 0.001<br>Month: < 0.001<br>Genotype x Month: < 0.001<br>NLPF vs. WT<br>3 months: 0.973<br>6 months: 0.534<br>9 months: 0.009<br>12 moths: < 0.001	2 way ANOVA post hoc holm-sidak Tukey's test
2B-CRT	Genotype: (1, 48) = 27.959<br>Month: (3, 48) = 8.718<br>Genotype x Month: (3, 48) = 8.243	Genotype: < 0.001<br>Month: < 0.001<br>Genotype x Month: < 0.001<br>NLPF vs. WT<br>3 months: 0.999<br>6 months: 0.460<br>9 months: < 0.001<br>12 moths: < 0.001	2 way ANOVA post hoc holm-sidak Tukey's test
2E		CA1: 0.015<br>CA3: 0.002<br>DG: 0.002	Mann-Whitney rank sum test
2H		CA1: 0.835<br>CA3: 0.615	Mann-Whitney rank sum test
3B	Genotype: (1, 98) = 120.307<br>Month: (3, 98) = 8.682<br>Genotype x Month: (3, 98) = 6.288	Genotype: < 0.001<br>Month: < 0.001<br>Genotype x Month: < 0.001<br>NLPF vs. WT<br>3 months: 0.195<br>6 months: < 0.001<br>9 months: < 0.001<br>12 moths: < 0.001	2 way ANOVA post hoc holm-sidak Tukey's test
3C	Genotype: (1, 98) = 61.971<br>Month: (3, 98) = 2.461<br>Genotype x Month: (3, 98) =	Genotype: < 0.001<br>Month: 0.067<br>Genotype x Month:	2 way ANOVA post hoc Holm-sidak Tukey's test

	2.210	0.092<br>NLPF vs. WT<br>3 months: 0.130<br>6 months: < 0.001<br>9 months: < 0.001<br>12 moths: < 0.001	
4A-3 months		25 ms: 0.319<br>50 ms: 0.610<br>100 ms: 0.584<br>200 ms: 0.537	Student's t-test
4A-6 months		25 ms: 0.440<br>50 ms: 0.195<br>100 ms: 0.689<br>200 ms: 0.482	Student's t-test
4A-9 months		25 ms: 0.355<br>50 ms: 0.226<br>100 ms: 0.306<br>200 ms: 0.820	Student's t-test
4A-12 months		25 ms: 0.630<br>50 ms: 0.856<br>100 ms: 0.645<br>200 ms: 0.862	Student's t-test
4C	Genotype: (1, 63) = 20.639<br>Month: (1, 63) = 1.381<br>Genotype x Month: (1, 63) = 0.378	Genotype: < 0.001<br>Month: 0.257<br>Genotype x Month: 0.769<br>NLPF vs. WT<br>1.5 months: 0.901<br>3 months: 0.028<br>6 months: 0.003<br>9 months: 0.161<br>12 moths: 0.020	2 way ANOVA post hoc holm-sidak Tukey's test
5B-CA1	Genotype: (1, 48) = 43.228<br>Month: (3, 48) = 0.356<br>Genotype x Month: (3, 48) = 0.918	Genotype: < 0.001<br>Month: 0.785<br>Genotype x Month: 0.440<br>NLPF vs. WT<br>3 months: < 0.001<br>6 months: 0.079<br>9 months: < 0.001<br>12 moths: < 0.001	2 way ANOVA post hoc holm-sidak Tukey's test
5B-DG	Genotype: (1, 47) = 24.976<br>Month: (3, 47) = 0.374<br>Genotype x Month: (3, 47) = 0.227	Genotype: < 0.001<br>Month: 0.772<br>Genotype x Month: 0.877<br>NLPF vs. WT<br>3 months: 0.003<br>6 months: 0.044<br>9 months: 0.034<br>12 moths: 0.014	2 way ANOVA post hoc holm-sidak Tukey's test
5B-CRT	Genotype: (1, 48) = 40.545	Genotype: < 0.001	2 way ANOVA post hoc holm-sidak Tukey's test

	Month: (3, 48) = 0.330<br>Genotype x Month: (3, 48) = 0.264	Month: 0.804<br>Genotype x Month: 0.851<br>NLPF vs. WT<br>3 months: 0.001<br>6 months: 0.022<br>9 months: 0.002<br>12 moths: 0.001	
5C-CA1	Genotype: (1, 48) = 25.489<br>Month: (3, 48) = 0.406<br>Genotype x Month: (3, 48) = 0.327	Genotype: < 0.001<br>Month: 0.749<br>Genotype x Month: 0.806<br>NLPF vs. WT<br>3 months: 0.029<br>6 months: 0.019<br>9 months: 0.001<br>12 moths: 0.048	2 way ANOVA post hoc holm-sidak Tukey's test
5C-DG	Genotype: (1, 47) = 16.071<br>Month: (3, 47) = 0.531<br>Genotype x Month: (3, 47) = 0.989	Genotype: < 0.001<br>Month: 0.664<br>Genotype x Month: 0.406<br>NLPF vs. WT<br>3 months: 0.022<br>6 months: 0.062<br>9 months: 0.003<br>12 moths: 0.499	2 way ANOVA post hoc holm-sidak Tukey's test
5C-CRT	Genotype: (1, 48) = 27.778<br>Month: (3, 48) = 0.542<br>Genotype x Month: (3, 48) = 1.599	Genotype: < 0.001<br>Month: 0.656<br>Genotype x Month: 0.202<br>NLPF vs. WT<br>3 months: 0.241<br>6 months: 0.049<br>9 months: < 0.001<br>12 moths: 0.002	2 way ANOVA post hoc holm-sidak Tukey's test
6A		3 months: 0.925<br>6 months: 0.029<br>9 months: 0.486<br>12 months: 0.340	3 months: Student's t-test<br>6 months: Mann-Whitney rank sum test<br>9 months: Mann-Whitney rank sum test<br>12 months: Student's t-test
6B		3 months: 0.593<br>6 months: 0.026<br>9 months: 0.025<br>12 months: 0.792	3 months: Student's t-test<br>6 months: Mann-Whitney rank sum test<br>9 months: Student's t-test<br>12 months: Mann-Whitney rank sum test
